# Inhibitory Bacterial Diversity and Mucosome Function Differentiate Susceptibility of Appalachian Salamanders to Chytrid Fungal Infection

**DOI:** 10.1128/aem.01818-21

**Published:** 2022-03-29

**Authors:** Randall R. Jiménez, Amy Carfagno, Luke Linhoff, Brian Gratwicke, Douglas C. Woodhams, Liana Soares Chafran, Molly C. Bletz, Barney Bishop, Carly R. Muletz-Wolz

**Affiliations:** a Center for Conservation Genomics, Smithsonian National Zoological Park and Conservation Biology Institute, Washington, DC, USA; b Department of Chemistry and Biochemistry, George Mason University, Manassas, Virginia, USA; c Center for Species Survival, Smithsonian National Zoological Park and Conservation Biology Institute, Washington, DC, USA; d Department of Biology, University of Massachusetts Boston, Boston, Massachusetts, USA; Michigan State University

**Keywords:** chytrid, microbiome networks, skin bacterial communities, skin mucus, skin peptides

## Abstract

Mucosal defenses are crucial in animals for protection against pathogens and predators. Host defense peptides (antimicrobial peptides, AMPs) as well as skin-associated microbes are key components of mucosal immunity, particularly in amphibians. We integrate microbiology, molecular biology, network-thinking, and proteomics to understand how host and microbially derived products on amphibian skin (referred to as the mucosome) serve as pathogen defenses. We studied defense mechanisms against chytrid pathogens, *Batrachochytrium dendrobatidis* (Bd) and *B. salamandrivorans* (Bsal), in four salamander species with different *Batrachochytrium* susceptibilities. Bd infection was quantified using qPCR, mucosome function (i.e., ability to kill Bd or Bsal zoospores *in vitro*), skin bacterial communities using 16S rRNA gene amplicon sequencing, and the role of Bd-inhibitory bacteria in microbial networks across all species. We explored the presence of candidate-AMPs in eastern newts and red-backed salamanders. Eastern newts had the highest Bd prevalence and mucosome function, while red-back salamanders had the lowest Bd prevalence and mucosome function, and two-lined salamanders and seal salamanders were intermediates. Salamanders with highest Bd infection intensity showed greater mucosome function. Bd infection prevalence significantly decreased as putative Bd-inhibitory bacterial richness and relative abundance increased on hosts. In co-occurrence networks, some putative Bd-inhibitory bacteria were found as hub-taxa, with red-backs having the highest proportion of protective hubs and positive associations related to putative Bd-inhibitory hub bacteria. We found more AMP candidates on salamanders with lower Bd susceptibility. These findings suggest that salamanders possess distinct innate mechanisms that affect chytrid fungi.

**IMPORTANCE** How host mucosal defenses interact, and influence disease outcome is critical in understanding host defenses against pathogens. A more detailed understanding is needed of the interactions between the host and the functioning of its mucosal defenses in pathogen defense. This study investigates the variability of chytrid susceptibility in salamanders and the innate defenses each species possesses to mediate pathogens, thus advancing the knowledge toward a deeper understanding of the microbial ecology of skin-associated bacteria and contributing to the development of bioaugmentation strategies to mediate pathogen infection and disease. This study improves the understanding of complex immune defense mechanisms in salamanders and highlights the potential role of the mucosome to reduce the probability of Bd disease development and that putative protective bacteria may reduce likelihood of Bd infecting skin.

## INTRODUCTION

The skin is the largest organ in vertebrates and upon pathogen exposure serves as a first line of defense against pathogens in vertebrates. Skin-associated mucosal defenses are derived from the resident microbes and their produced compounds as well as those produced by the host, such as antimicrobial peptides (AMPs, sometimes designated defense peptides) (1). In addition to other mucosal defense factors, host-associated bacterial communities consist of many interacting species, and are known to contribute to pathogen defense, maintenance of host health (2), and disease dynamics (3). Likewise, AMPs are essential components of innate defenses in animals with a broad spectrum of activities, as they may alter resistance to pathogenic infections and/or facilitate bacterial growth, and their antimicrobial ability is microorganism-dependent (4–6). Together, all these compounds make up the skin mucosal ecosystem, known as the mucosome, that protects hosts against invading pathogens (7). Understanding the foundations of anti-pathogen skin function and interactions among the host microbiota, host peptides, and pathogens is relevant to understand adaptations of wild vertebrate populations in response to pathogens.

Infectious diseases are increasingly recognized as an important factor governing wildlife population dynamics (8, 9). For instance, chytridiomycosis is a skin disease caused by chytrid fungal pathogens *Batrachochytrium dendrobatidis* (Bd) and *B. salamandrivorans* (Bsal) that impacts amphibians worldwide (10, 11). However, amphibians differ widely in their susceptibility to these pathogens (5, 12). Skin microbiomes, skin peptide repertoires, and their collective mucosome properties are suggested as the primary barriers protecting amphibians from these pathogenic chytrids (5, 7, 13–16). Similar processes may also affect disease ecology in other wildlife systems, including white-nose syndrome in bats (17) and snake fungal disease (18).

Innate immunity protects vertebrates against infectious pathogens/diseases through distinct components, such as host-associated bacteria and AMPs. A considerable amount of research has elucidated the contribution of the skin microbiome in the defense against pathogens (17, 18), particularly Bd on amphibians (1, 7, 19). Many of the bacteria on amphibian skin show broad-spectrum antimicrobial activity with the ability to inhibit Bd growth (19–21). Further, the relative abundance of Bd-inhibitory bacteria in the host skin bacterial community has been shown to affect Bd infection in hosts by potentially diminishing the infection (22). Concerning AMPs, these defensive molecules are considered reliable predictors of natural resistance against pathogens (4). Many amphibian peptides and peptide mixtures have been shown to inhibit different pathogens, including Bd (14, 15, 23). Voyles et al. (5) found that neotropical frogs had stronger Bd-inhibitory activity of AMPs after a major disease epizootic event, indicating shifts in host immune defenses in response to pathogen pressure. The skin mucosome serves as an effective predictor of Bd and Bsal susceptibility (7, 16), suggesting that the shared contribution of bacteria and AMPs in the mucosome collectively explain why some species are more susceptible than others.

It is clear that a more detailed understanding is needed of the interactions between the host and its microbiome functioning in pathogen defense. The production of antifungal metabolites by symbiotic bacteria may be a consequence of specific bacterial interactions within communities. For instance, the Bd-inhibitory bacterial metabolites violacein produced by Janthinobacterium lividum (24) is a by-product of bacterial competition (25). Microbiome networks can be used to detect possible associations through co-occurrence of microorganisms and the influence of bacterial interactions on the host health (26, 27). In addition, network analysis can predict highly associated taxa in a microbial network known as hub taxa (28, 29). These hubs have been suggested as potential keystones taxa: ecologically important bacteria responsible for maintaining community structure and adequate functionality of the system (30).

Our study system uses four wild salamander species (*Plethodon cinereus*, *Eurcyea bislineata*, *Desmognathus monticola* and *Notophthalmus viridescens*), that commonly occur throughout the Appalachian region of the eastern US and differ in their susceptibility to the chytrid pathogens (Table 1). The chytrid Bd is endemic in this region, and Bsal is currently absent from the US (12, 31). There is a risk of Bsal or a novel Bd strain being introduced into the US and causing widespread population declines and extinctions in this unique hot spot of salamander biodiversity (32). The aims of this study were to (i) determine Bd infection prevalence and intensity among salamander species, (ii) quantify the skin mucosome function (i.e., ability of skin secretions to kill Bd and Bsal zoospores) among salamander species and understand its relationship to Bd infection in the wild, (iii) characterize skin bacterial communities across salamander species and environments, particularly the relationship between richness and relative abundance of putative Bd-inhibitory bacteria with Bd infection, (iv) assess the contribution of putative Bd-inhibitory bacteria to the network structure and their associations within the network, and (v) explore the presence of candidate antimicrobial peptides in *P. cinereus* and *N. viridescens*. We expected that as mucosome function increased Bd infection prevalence and intensity would decrease (7, 16). We expected that as putative Bd-inhibitory bacteria increased in richness and abundance Bd infection prevalence and intensity would decrease (13, 33). Finally, we hypothesized that putative Bd-inhibitory bacteria are important in microbiome network structure (i.e., the most interactive ASVs, “hub taxa”) (34). Given the importance of innate immune defenses on vertebrates, the understanding of the immune mechanisms in amphibians with advanced approaches and technologies will lead us to the discovery of novel aspects in vertebrate’s immunity.

**TABLE 1:** The four focal salamander species and their categorized chytrid susceptibility^*a*^

Common name	Species name	Bd susceptibility	Bsal susceptibility	Reference
Red-backed salamander	*Plethodon cinereus*	Resistant	Tolerant	83 – 85
Two-lined salamander	*Eurcyea bislineata*	Tolerant	Susceptible	83, 86, 87
Seal salamander	*Desmognathus monticola*	Tolerant	Resistant	83, 87–89
Eastern newt	*Notophthalmus viridescens*	Tolerant/susceptible	Lethal	83, 90, 91

a See Van Rooij et al. (82) for the concepts of resistant, tolerant and susceptible used in this study.

## RESULTS

**FIG 1:**
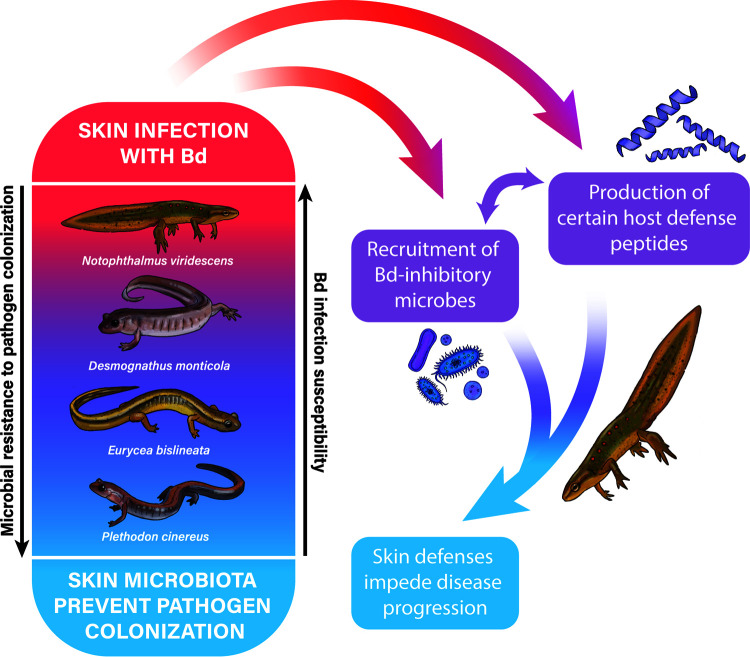
Hypothetical model of innate immune defenses against the chytrid pathogen Bd linked to differential Bd susceptibility in Appalachian salamanders. Illustration elaborated by Nina McDonnell.

Mucosome-killing ability against Bd and the diversity of Bd-inhibitory bacteria were linked to differential susceptibility in our samples of Appalachian salamanders (Fig. 1). We present those results here.

### Bd infection prevalence and intensity in the wild and mucosome-killing ability against Bd and Bsal.

Bd infection prevalence differed significantly among species (Table 2). No individual of *P. cinereus* was infected and 16% of individuals of *E. bislineata* were infected with Bd. The species *D. monticola* and *N. viridescens* showed the highest Bd infection prevalence among our study species, 45% and 56% infected, respectively. We also found that Bd infection intensity differed significantly among species. Individuals of *N. viridescens* showed the highest Bd infection intensity followed by *E. bislineata* and *D. monticola* (Table 2).

**TABLE 2:** Bd infection prevalence and intensity information for four salamander species^*a*^

Species	N	Bd prevalence Mean (95% CI)	Infection intensity (Bd ZGEs) Mean ± SD
*P. cinereus*	35	0.0 (0.0 – 0.10)	0.0
*E. bislineata*	12	0.16 (0.02 – 0.48)	106 ± 118
*D. monticola*	11	0.45 (0.16 – 0.76)	8.5 ± 4.4
*N. viridescens*	25	0.56 (0.35 – 0.76)	252 ± 610

a Infection intensity of Bd is from infected individuals only. ZGEs, zoospore genomic equivalents.

**FIG 2:**
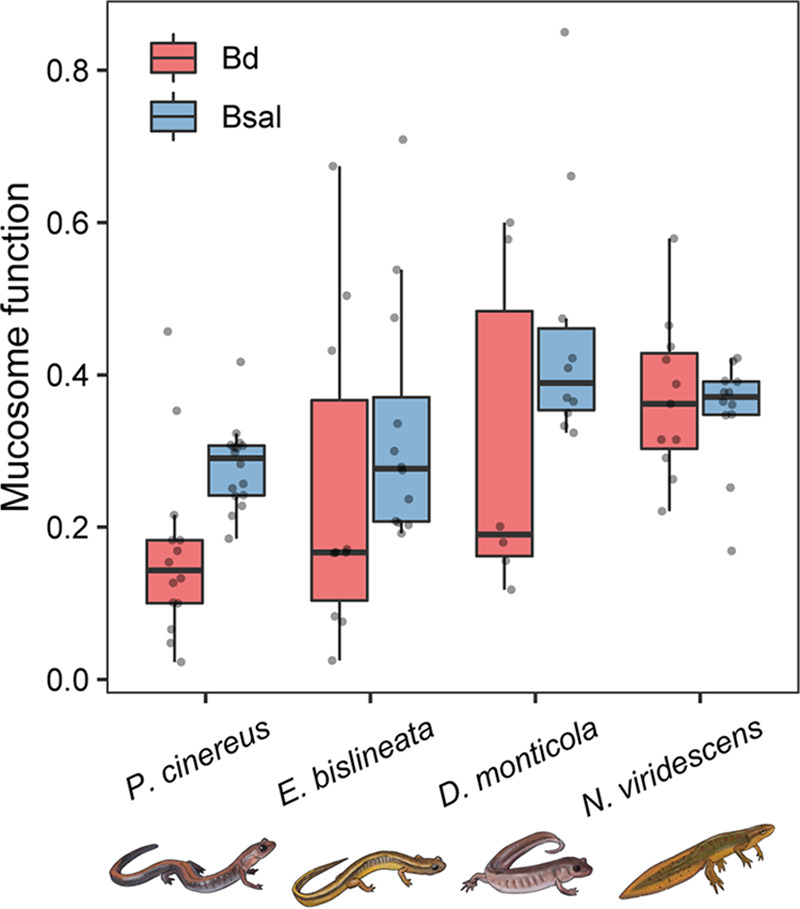
Mucosome function (measure of the pathogen-killing ability of skin mucus samples) against the chytrid pathogens Bd and Bsal among four salamander species. Each gray point represents an individual sample.

Mucosome function against Bd and Bsal differed among species (Fig. 2, GLM: Bd χ^2^ = 10.59, *P* = 0.01, Bsal χ^2^ = 13.72, *P* = 0.003), with *P. cinereus* and *N. viridescens* differing in mucosome function (Fig. 2, Bd and Bsal *P* < 0.05). When mucosome function was evaluated for Bd and Bsal within the same salamander species, we found that the mucosome of *P. cinereus* had a lower ability to kill Bd than Bsal (Fig. 2, *t* test: t = −350, *P* = 0.002). The other species showed similar mucosome-killing function for both pathogens (Fig. 2).

**FIG 3:**
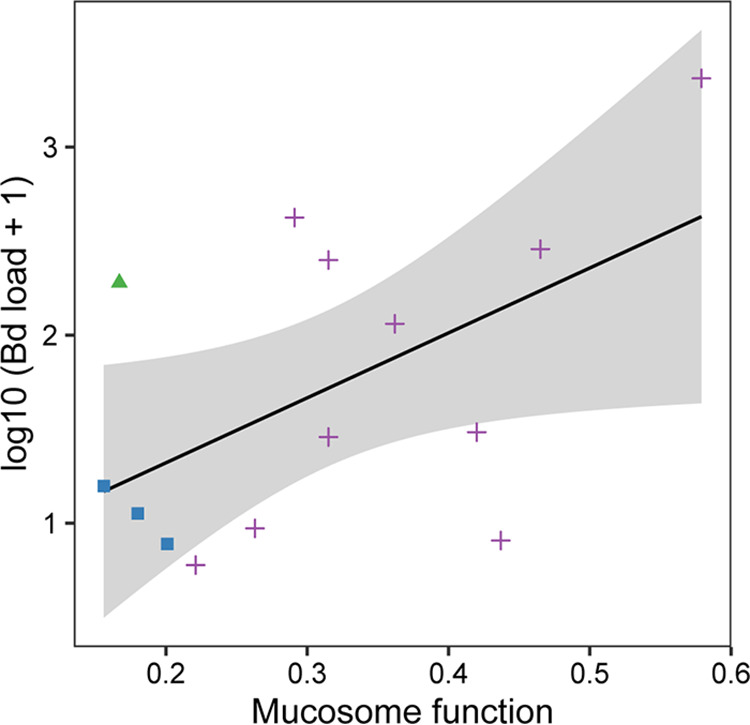
Relationship between Bd infection intensity and mucosome function (measure of the pathogen-killing ability of skin mucus samples) on salamanders. Species samples were pooled to performed hurdle model. The continuous lines indicate the predicted fit and the shaded areas are 95% confidence intervals. Samples were coded by color and shape to observed species samples: green triangles = *E. bislineata*, blue squares = *D. monticola* and purple cross = *N. viridescens*.

We did not find an association between mucosome function and Bd infection prevalence (hurdle binomial model: *F *= 1.92, *P* = 0.18) across salamander species. However, we observed a relationship between mucosome function and Bd infection intensity (hurdle count model: χ^2^ = 7.27, *P* = 0.01). Individuals with higher mucosome function showed higher Bd infection intensity (Fig. 3) across salamander species. Likewise, a significant positive association was found between mucosome function and Bd infection intensity in newts only (negative binomial: *z *= 2.17, *P* = 0.03; Fig. S1), suggesting that newts may be driving the association between mucosome function and Bd infection intensity observed across salamander species (Fig. 3).

### Microbiome characterization in wild salamanders.

**FIG 4:**
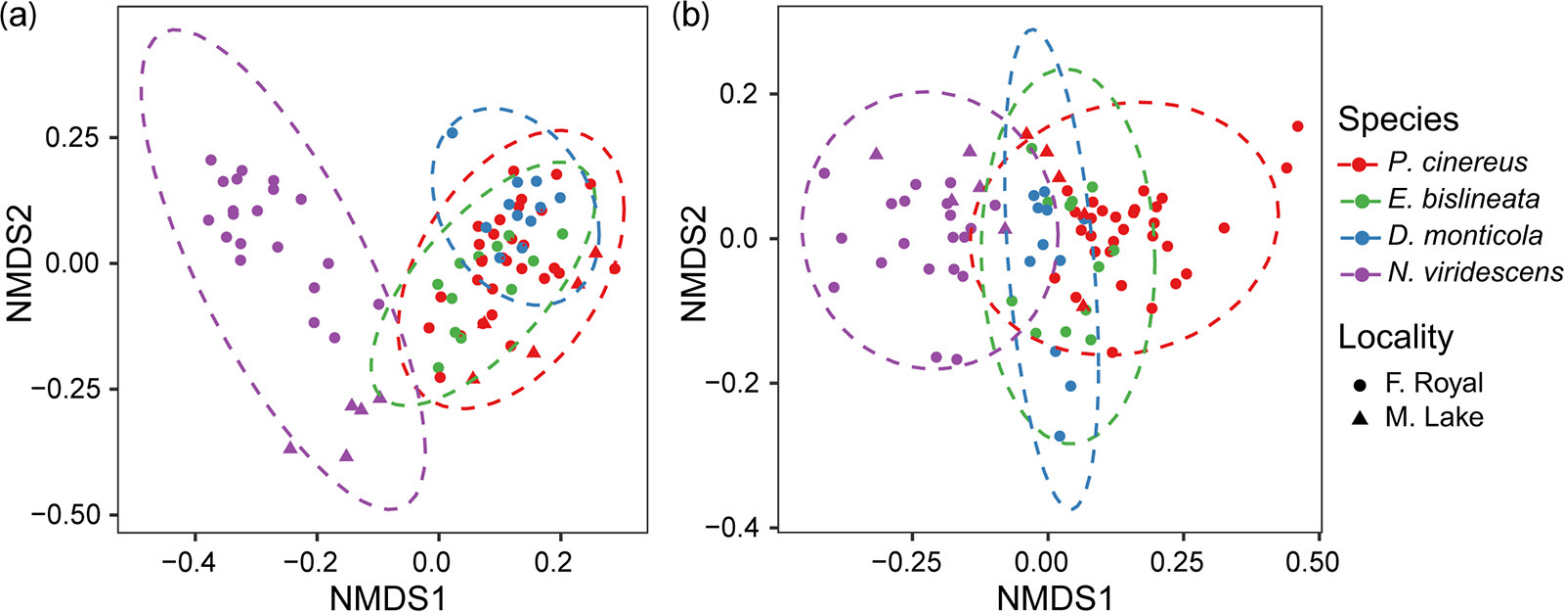
Non-Metric Multidimensional Scaling plots (NMDS) based on the (a) unweighted UniFrac and (b) weighted UniFrac showing bacterial beta diversity across salamander species sampled at two localities. Each point represents the bacterial skin community of an individual; point color indicates host species and shape indicates sampling locality. Ellipses show 95% confidence intervals (95% CIs) of each host species.

Salamander species had distinct skin microbiomes. We observed differences in bacterial composition at family and genus level (detailed information in supplemental results; Fig. S2). Host species had a strong effect on microbiome structure, while environment had a smaller effect. Bacterial ASV richness and phylogenetic diversity differed among host species (Table S2, Fig. S3, GLM: ASVs richness χ^2^ = 15.56, *P* = 0.001 and PD χ^2^ = 9.04, *P* = 0.013) and locality (Table S2, *P* < 0.05). *Desmognathus monticola* had higher ASV richness and phylogenetic diversity than *E. bislineata*, *P. cinereus* and *N. viridescens* (Fig. S3a-b, *P* < 0.05). Bacterial community composition differed among host species (PERMANOVA: unweighted UniFrac R^2^ = 0.28, *P* = 0.001, weighted UniFrac R^2^ = 0.45, *P* = 0.001; Table S3), and locality (PERMANOVA: unweighted UniFrac: R^2^ = 0.06 *P* = 0.001, weighted UniFrac: R^2^ = 0.03, *P* = 0.001; Table S3). All species differed in bacterial community presence-absence composition (Pairwise PERMANOVA: unweighted UniFrac: *P* < 0.05, Table S4). All species differed in bacterial community abundance-weighted composition (Pairwise PERMANOVA: weighted UniFrac: *P* < 0.05), except between *E. bislineata* with *D. monticola* (Pairwise PERMANOVA: weighted UniFrac: *P* = 0.88) (Fig. 4, Table S4).

### Characterization of putative Bd-inhibitory bacteria in wild salamanders.

**FIG 5:**
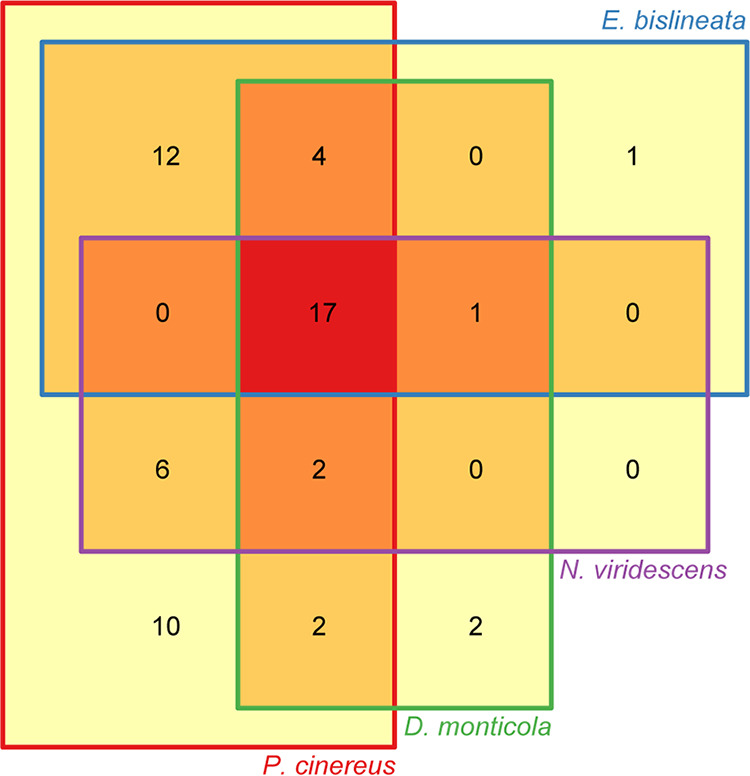
Venn diagram showing putative Bd-inhibitory ASVs across four salamander species.

Among the four salamander species, we found 57 putative Bd-inhibitory bacterial ASVs in their skin microbiomes (Table S5). The salamander *P. cinereus* had the highest number of these putative Bd-protective bacterial strains (53 ASVs), while *N. viridescens* had the lowest number (26 ASVs) (Fig. 5). A total of 17 ASVs with putative Bd-inhibitory activity were present in all four species, and *P. cinereus* and *E. bislineata* showed the highest overlap (12 ASVs) (Fig. 5). The putative Bd-inhibitory ASVs assigned to the genera Pseudomonas were present in all species and with the highest abundance (Table S5).

### Relationship between putative Bd-inhibitory bacteria diversity, Bd infection and mucosome function.

**FIG 6:**
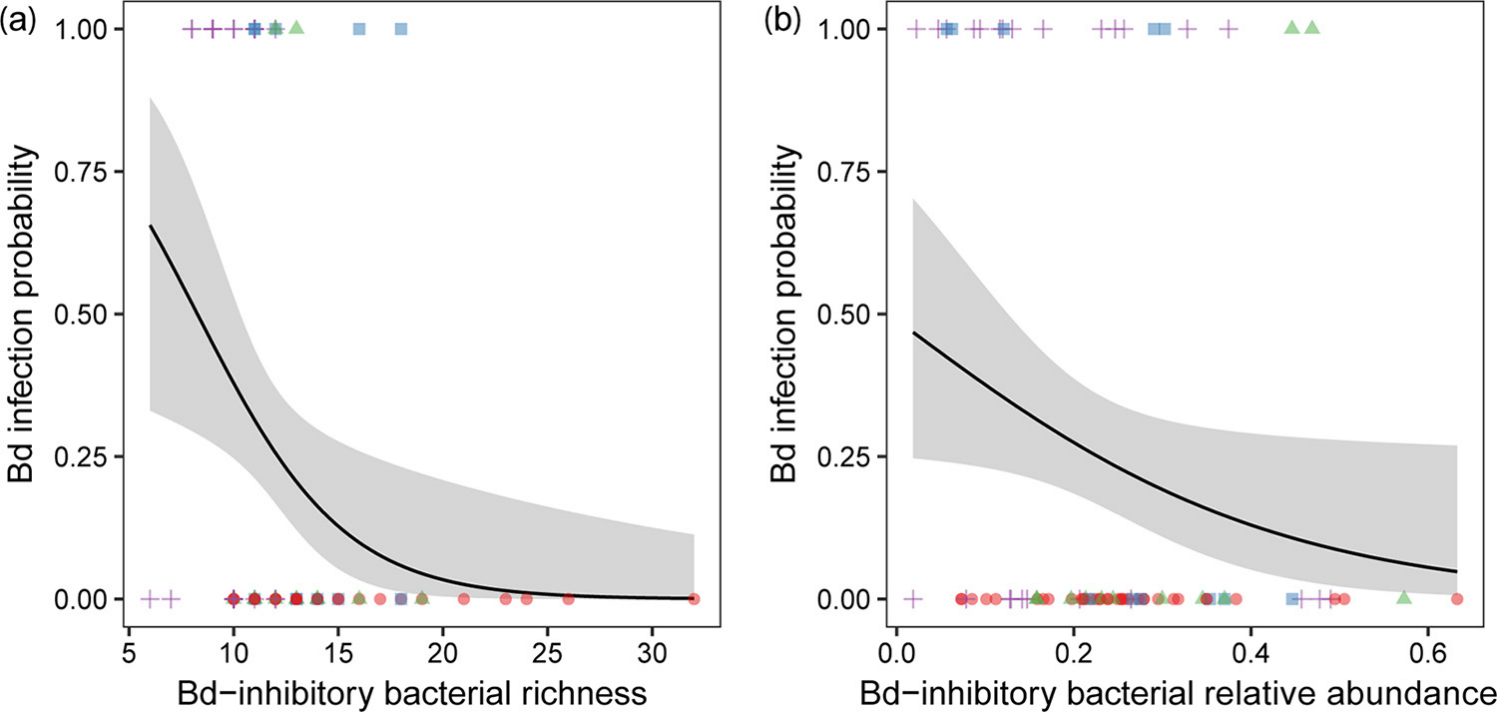
Relationship of Bd infection prevalence with putative Bd-inhibitory bacterial richness (a) and relative abundance (b) on salamanders. The continuous lines indicate the predicted fit and the shaded areas are 95% confidence intervals. Samples were coded by color and shape to observed species samples: red circles = *P. cinereus*, green triangles = *E. bislineata*, blue squares = *D. monticola* and purple cross = *N. viridescens*.

There was a significant association between putative Bd-inhibitory bacterial richness and the Bd infection prevalence (hurdle binomial model: *F *= 8.73, *P* = 0.004) and between the relative abundance of Bd-inhibitory bacteria and the Bd infection prevalence (hurdle binomial model: *F *= 4.44, *P* = 0.04). No associations were detected with Bd infection intensity (richness hurdle count model: *F *= 1.18, *P* = 0.27; relative abundance hurdle count model: *F *= 0.53, *P* = 0.45). Bd infection prevalence decreased as the richness of the Bd-inhibitory bacteria increased (Fig. 6a) and as the relative abundance of the Bd-inhibitory bacteria increased (Fig. 6b). No significant associations were found of ASV bacterial richness of whole community with Bd infection prevalence (hurdle binomial model: *F *= 1.59, *P* = 0.21) and intensity (hurdle count model: *F *= 0.27, *P* = 0.60).

There were no significant relationships of mucosome function with Bd-inhibitory bacterial richness (GLM: χ^2^ = 1.15, *P* = 0.28) or relative abundance (GLM: χ^2^ = 1.53, *P* = 0.21) when considering both Bd-infected and uninfected individuals or for Bd-infected individuals only (ASV richness: GLM: χ^2^ = 1.27, *P* = 0.25; relative abundance: GLM: χ^2^ = 0.20, *P* = 0.65). Likewise, no significant relationship was found between mucosome function and ASV bacterial richness of whole community when taking into account both Bd-infected and uninfected individuals (GLM: χ^2^ = 0.10, *P* = 0.74) or for Bd-infected individuals only (GLM: χ^2^ = 0.15, *P* = 0.70).

### Role of putative Bd-inhibitory bacteria as key bacteria in network structure.

**FIG 7:**
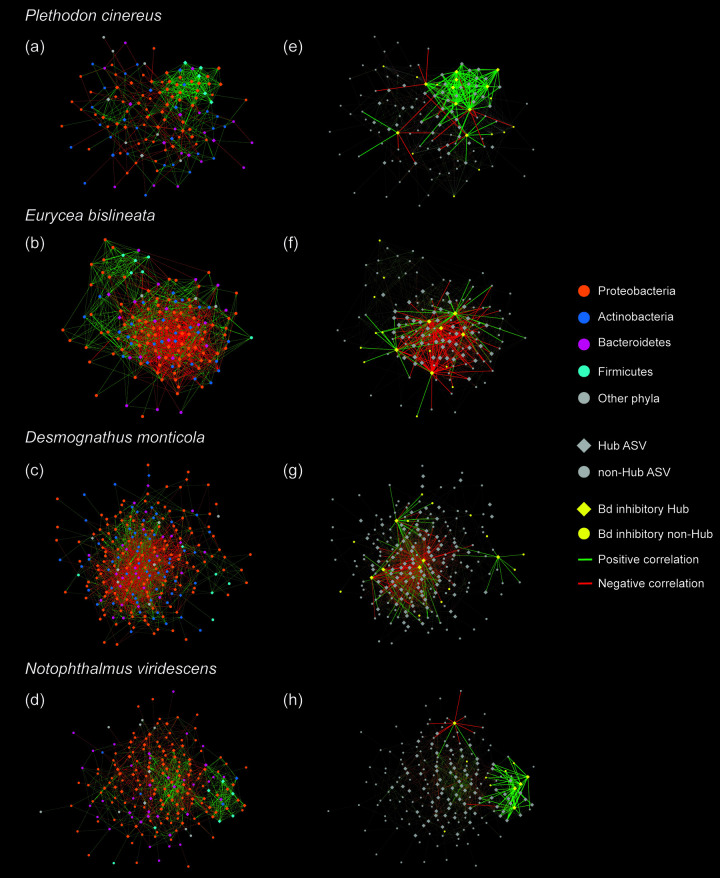
Co-occurrence networks of the skin bacterial communities per salamander species. Nodes represent ASVs. Edges represent significant correlations. Networks on the left side (a, b, c, d) show all nodes colored by bacterial phylum and the nodes that represent hub and nonhub ASVs are shape coded. Networks on the right side (e, f, g, h) show the nodes that represent hub and nonhub ASVs and putative Bd inhibitory ASVs and are color and shape coded. Only positive and negative correlations for Bd inhibitory hubs are highlighted in networks on the right side (e-h).

All salamander species had tightly connected skin microbiome networks, in which most bacteria were associated with every other bacteria through a relatively short path (small world networks: Layeghifard et al., [26]). Across all salamander species, skin microbiome networks showed a signature of bacteria commonly associated with bacteria from phyla Proteobacteria and Actinobacteria, and with limited clustering within networks (Fig. 7a to d). We detected a similar proportion of hub taxa among salamander species (*χ*^2^ = 1.65, *P* = 0.648; Fig. 7a to d), but a different proportion of putative Bd-inhibitory hub bacteria among species (*χ*^2^ = 7.59, *P* = 0.04), which was linked to their Bd susceptibility: *P. cinereus*, which had the lowest susceptibility to Bd, had the highest proportion of hubs that are associated with Bd-inhibition (7.8%; 10 of 129 bacteria defined as nodes), followed by *E. bislineata* (4.1%; 6/146), and *D. monticola* (2.5%; 6/238) and *N. viridescens* (2.5%; 5/201) (Fig. 7e to h, Table 3). Across salamander species, the Bd-inhibitory hub bacteria involved in network structure belonged to the genera Acinetobacter, Pseudomonas, *Microbacterium* and *Curtobacterium*.

Bd-inhibitory hub bacteria in the networks commonly had positive correlations with other bacteria indicative of a fine-scale aggregation, relevant ecological relationships, similar niches, and even potentially obligate partnerships. We observed a different proportion of associations with Bd-inhibitory hub bacteria involved among salamander species (*χ*^2^ = 130.95, *P* < 0.0001). *Plethodon cinereus* had the highest proportion of positive correlations with Bd-inhibitory hub bacteria involved (89%), compared to the other salamander species, indicating that *P. cinereus* may be host to a bacterial community of highly connected Bd-inhibitory bacteria (Table 3). For associations of putative Bd-inhibitory bacteria as hubs only, we found that the Bd-inhibitory hub bacteria were almost always positively correlating with one another for all salamander species, except for *E. bislineata*, suggesting positive feedbacks among these Bd-inhibitory bacteria (Fig. 7e to h). Similar pattern of associations was observed with all Bd-inhibitory bacteria (hub and nonhub together) in the networks (Fig. 7e to h). Further, we observed a distinct proportion of correlations of putative Bd-inhibitory bacteria as hubs only across salamander species (G-test = 15.65, *P* = 0.001): *N. viridescens* showed the highest proportion of positive associations (100%), followed by *P. cinereus* (95%), *D. monticola* (85%) and *E bislineata* (53%) (Table 3). Interestingly, we found that all our culturable Bd-inhibitory bacteria did not inhibit the growth of one another providing support for the positive associations we observed of Bd-inhibitory hub bacteria with other bacteria (Table S6). Lastly, we examined correlations among hub bacteria, regardless of their putative Bd-inhibition properties. Salamander species differed in the proportion of hub bacteria they hosted in their skin microbiome (*χ*^2^ = 423.77, *P* < 0.0001). *P. cinereus* had more positive associations compared to the other salamanders indicating that the bacterial communities of this species may be more connected and suggesting that *P. cinereus* may be host to a unique community of highly connected protective bacteria such as the identified Bd-inhibitory hub bacteria (Table 3).

**TABLE 3:** Summary of network structure among salamander species related to putative Bd-inhibitory ASVs^*a*^

Variable	*P. cinereus*	*E. bislineata*	*D. monticola*	*N. viridescens*
Total nodes	129	146	238	201
Total hub bacteria^*b*^	55 (0.43)	52 (0.36)	97 (0.41)	82 (0.41)
Bd inhibitory hub bacteria^*c*^	10 (0.08)	6 (0.04)	6 (0.02)	5 (0.02)
Bacterial correlations linked to Bd-inhibitory hub bacteria^d^				
Positive	143 (0.89)	48 (0.32)	72 (0.56)	64 (0.86)
Negative	17 (0.11)	102 (0.68)	57 (0.44)	10 (0.14)
Bacterial correlations between Bd-inhibitory hub bacteria only^d^				
Positive	20 (0.95)	9 (0.53)	6 (0.85)	12 (1.00)
Negative	1 (0.05)	8 (0.47)	1 (0.15)	0 (0.00)
Bacterial correlations linked to all hub bacteria^d^				
Positive	390 (0.80)	367 (0.35)	576 (0.39)	565 (0.66)
Negative	95 (0.20)	669 (0.65)	893 (0.61)	290 (0.34)

a Data in parenthesis represent the proportion.

b Total hub bacteria proportion: total hub bacteria / total nodes.

c Bd inhibitory hub bacteria proportion: number of Bd inhibitory hub bacteria / total nodes.

d Bacterial correlation proportions: number of correlations (positive or negative) / total number of correlations.

### Peptide characterization in wild salamanders.

We identified 13,138 total features, of which we identified 1,212 total candidate AMPs (features with gas-phase charge between +3 to +8 and mass ≥ 1000). Between salamander species, we detected a comparable number of candidate AMPs between the nonstimulated individuals of both species (*N. viridescens*:46–56 features, *P. cinereus*:39–42 features), while for acetylcholine-induced individuals we observed a higher number of candidate AMPs on *P. cinereus* than on *N. viridescens* (∼3 times higher) (Table 4). A substantial number of candidate AMPs were detected on one acetylcholine-induced *N. viridescens* (LEEP-17), though such features tended to be lower abundance than those detected in the acetylcholine-induced *P. cinereus* samples. Specifically, only five candidate AMPs from sample LEEP-17 exhibited normalized feature area ≥ 1E6, whereas approximately 10 times as many candidates AMPs were observed at this relative abundance in the acetylcholine-induced *P. cinereus*.

**TABLE 4:** Summary of peptide feature detection among two salamander species

Sample ID	Peptide induction	# Features	# Identified features	# Candidate AMPs (charge +3 to +8; Mass ≥ 1000)	# Identified candidate AMPs (charge +3 to +8; Mass ≥ 1000)
*N. viridescens*					
LEEP13	Soak	1195	39	46	0
LEEP15	Soak	1633	61	56	0
LEEP17	Acetylcholine	1841	83	117	4
LEEP20	Acetylcholine	1187	21	53	0
*P. cinereus*					
POSH21	Soak	1269	29	42	0
POSH22	Soak	1586	64	39	0
POSH24	Acetylcholine	1903	93	404	27
POSH25	Acetylcholine	2524	206	455	68

We found a low proportion of features with high-scoring identifications assigned by PEAKS (Table 4). Sequences were further analyzed with BLAST, and six peptides from *P. cinereus* matched vertebrate protein sequences. The six peptides showed low complexity and were glycine-rich, and none of the alignments were to regions of proteins annotated with known antimicrobial function. The limited PEAKS identifications are not necessarily indicative of lack of peptides with antimicrobial function, but a potential lack of completeness of the database.

**FIG 8:**
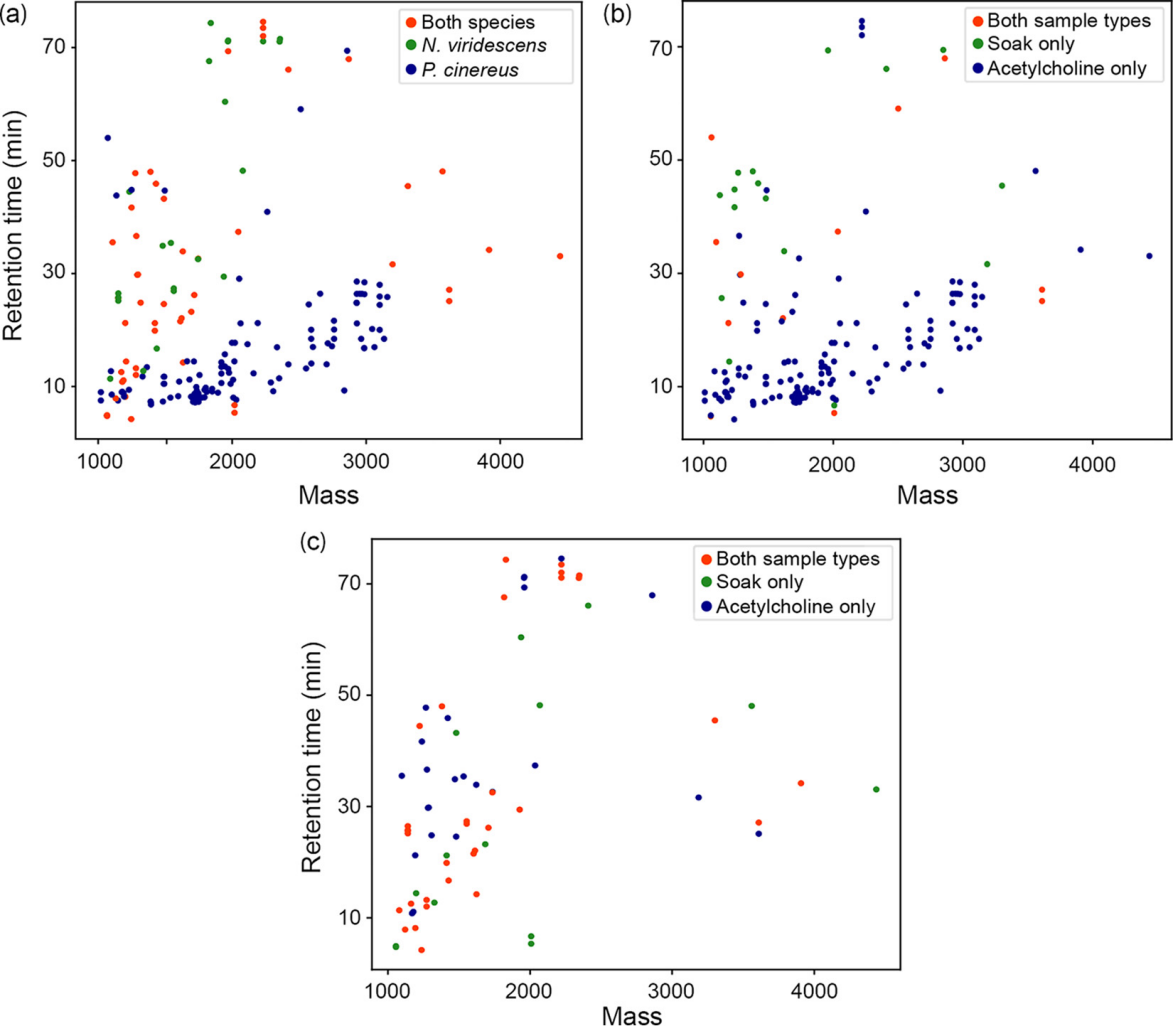
Features with mass ≥ 1000 and gas-phase charge between +3 to +8 detected in (a) all salamander samples, (b) *P. cinereus* (POSH samples) and (c) *N. viridescens* (LEEP samples). To better visualize shared features, features with detection in only 1 sample are not included in plots.

In the absence of a high-scoring sequence identification, we used feature-based label free quantification to evaluate differences in peptide features among samples. Though it is not possible to definitively conclude that a feature corresponds to a peptide, rather than a contaminant, matching features between samples and applying filtration criteria are nonetheless valuable for semiquantitative comparison of putative peptide profiles between samples. The 13,138 features detected in all salamander samples collapsed to 7,999 feature groups; 2,651 features groups were detected in at least two salamanders, and 638 feature groups were detected in at least four salamanders. The 1,212 candidate AMPs collapsed into 986 candidate AMP feature groups; 182 feature groups were detected in at least two salamanders (Fig. 8A to C; Table S7), and 11 feature groups were detected in at least four salamanders. Seven candidate AMP feature groups were detected in at least two samples of each species, and two of these seven exhibited relatively high abundance (normalized feature area ≥ 1E6) in at least one sample (Table S7). We observed a higher number of feature groups for *P. cinereus* compared to *N. viridescens* (Fig. 8A; TableS7).

## DISCUSSION

We show that amphibian skin-associated mucosal immunity plays a role in amphibian-pathogen dynamics, which vary among co-occurring Appalachian salamander species. In our study system, the eastern newts we sampled were aquatic adults inhabiting a pond within 200 m of the forested riparian site where the other three species were sampled at the Front Royal, VA locality. Some of the observed differences between newts and the other three species may be a reflection of fully aquatic versus semi-aquatic/terrestrial environments. However, the differences among red-backed, two-lined and seal salamanders cannot be solely explained by environmental differences. Particularly between red-backed and two-lined salamanders and between two-lined and seal salamanders that were often found under the same cover object (e.g., log, rock) or within 1 m in the more riparian areas of the site. We demonstrate that the variability in chytrid susceptibility of these Appalachian salamanders is not solely a reflection of their environment or exposure to the pathogen but is more a reflection of the innate defenses each species uniquely possesses to mediate infection and disease.

Observed differences in Bd prevalence reflected presumed Bd susceptibility across salamander species (Table 1), with red-back salamanders the least Bd susceptible, eastern newts the most susceptible, and two-lined salamanders and seal salamanders displaying intermediate susceptibility. It is likely that Bd has been present in the eastern US since at least 1978 (35), but potentially dating back to the late 1800s (36). Over the decades of potential exposure to Bd, Appalachian salamanders may have developed or utilized preexisting innate and adaptive immune mechanisms such as microbes (19), AMPs (37), or MHC (38) to tackle infection and disease.

Innate immune defense mechanisms are highly relevant first line of defenses in vertebrates against pathogens (39). We found that eastern newts with the highest pathogen burdens, but no evident symptoms of disease, had more robust mucosome function (shared bacterial and host-derived antifungal products). We found that red-backed salamanders with no signs of infection (even though co-occurring with infected hetero-specific individuals) contrarily had less robust mucosome function, but a more robust protective microbiome (Bd-inhibitory bacterial richness, Bd-inhibitory hub bacteria and positive microbial associations). Two-lined and seal salamanders had mixed defense strategies, including similar mucosome function, but a higher number of Bd-inhibitory bacteria on two-lined salamanders.

Salamanders with higher Bd infection loads showed higher mucosome function, but hosted few Bd-inhibitory bacteria (e.g., eastern newts). Furthermore, our results show a potential relationship between mucosome function and Bd intensity, which should be viewed with caution due to the small sample size of Bd infected animals for two-lined salamanders and seal salamanders. Indeed, we found that newts alone showed a relationship with mucosome function, indicating that this relationship may be species-specific to newts. Based on these results, we suspect that AMPs could be playing a role in the mucosome function to mediate chytrid growth and consequently protect against disease development, particularly on our highly infected and most Bd susceptible animals (i.e., Bd infection could be inducing the production of certain host defense peptides across hosts). This is congruent with Smith et al. (16), which demonstrated that peptides are important compounds providing a strong effect of the mucosome in salamanders not the bacteria and their metabolites. We found that eastern newts produced fewer peptides than red-backed salamanders suggesting that having many different peptides does not necessarily lead to strong mucosome function, but effectiveness and quantity of AMPs may be more important than their diversity. AMPs are known to have varied degrees of specificity and co-occurring AMPs can have synergistic activity resulting in more effective killing (4). We suggest that specific AMPs, regardless of their number, are providing stronger activity to mediate against chytrids (4, 40). However, we cannot determine the role of AMPs mediating the growth of chytrids in this study; therefore, additional analysis would be needed to determine their Bd inhibitory effect. Future work toward a better understanding of the role of different AMPs in the salamanders mucosome function is needed to achieve robust conclusions.

The observation that higher proportions of Bd-inhibitory taxa were associated with lower infection probability, suggests that these bacteria may be acting in a way that prevents Bd establishment (e.g., specific bacteria and/or bacterial interactions producing compounds that inhibits Bd). Although fewer Bd-inhibitory bacteria were found on more Bd-infected individuals, this does not necessarily imply that they are not playing a role in Bd defense. It is possible that AMPs could be interacting with specific Bd-inhibitory bacteria (and their metabolites) to help mediate Bd infection intensity (41). A recent study showed that peptide Brevinin-1Ma from the frog *Rana sierrae* possess the capacity to inhibit the growth of Bd and Bsal and enhances the growth of bacteria that produce bacterial metabolites against Bd (e.g., Janthinobacterium lividum, Chryseobacterium ureilyticum, and Pseudomonas sp.; Woodhams et al., [6]). It is also likely that bacteria secrete compounds during infection that induce the expression of some AMPs in hosts that could help mediate pathogen infection (42). For instance, the high proportion of positive interactions related to Bd-inhibitory hub bacteria in eastern newts, could be a mechanism whereby the bacteria produce compounds that not only help mediate the development of chytridiomycosis but also trigger the production of certain AMPs in hosts to help eliminate Bd. Our data suggest that the initial composition of skin bacteria is important in resisting infection (e.g., in red-backed salamanders), and that upon infection, some salamanders (e.g., eastern newts) could increase protective mucosal secretions that may act directly to inhibit chytrid pathogens or indirectly by functioning to facilitate specific Bd-inhibitory hub bacteria (Fig. 1). Although these explanations are intriguing and very plausible, further research is needed.

Unfortunately, we were unable to identify most candidate AMPs in our data set. The low proportion of identifiable features may be indicative of limitations in the searchable protein database, since the same sample preparation protocol was used to successfully detect known antimicrobial peptides and their precursors from Xenopus laevis, including magainin, PYLa/PGLa A, and xenopsin (data not shown). It is important to highlight that our limited identifications of peptides are not necessarily indicative of lack of AMPs in the sample. Identification of intact endogenous peptides is further complicated by lack of enzyme-based database search restriction (43). Amphibians have hyper diverse AMP assemblages compared to other vertebrates (4), and coupled with few genomic databases for amphibians, it is likely that salamander peptide repertoires represent many undiscovered AMPs.

An interesting finding is that red-backed salamanders that were not infected with Bd and host to a diversity of Bd-inhibitory bacteria and candidate AMPs had the least effective mucosome compared to other species. The lower mucosome activity in the red-backed salamanders could indicate that these animals, while host to these defense components, had less active anti-chytrid components in their mucus at the time of sampling because none were infected. However, the set of associated Bd-inhibitory bacteria suggest that these animals have a broad protective bacterial pool primed to mitigate Bd growth and establishment through the production of antifungal compounds. Therefore, it is possible that mucosome samples taken through water baths in our samples are missing much of the bacterial metabolites produced. Recent studies show that a greater richness and abundance of protective bacteria can better suppress Bd than a more limited diversity, and that high interactions of these type of bacteria may lead to resistance to chytrid infections (13, 33, 34). It is also possible that the difference of mucosome function between salamanders could be attributed differences in the speed of action of AMPs to kill Bd/Bsal zoospores in the assays. Taken together, our data suggest the existence of distinct innate defense mechanisms involved in resistance to the chytrid fungus in Appalachian salamanders, and the efficacy of these defenses varies among these animals.

Distinct bacterial communities were observed among salamander species, among environments and depending on Bd infection status. These could be explained by different host traits and external factors (44, 45). It is likely that AMPs associated with mucosal surfaces are driving the composition of the microbial communities and controlling the extent of colonization (46, 47). For instance, Davis et al. (48) found that host skin peptides are contributing to the maintenance of an amphibian species-specific microbial repertoire. *Plethodon cinereus* show the highest number of potentially beneficial bacteria, which means that these species possess a wide stock of bacteria with the ability to protect against Bd and Bsal (21, 44). Fungal micro-eukaryotes, such as chytrids, have been found to be very abundant and diverse in soils (49). It is likely that red-backed salamanders, the sole fully terrestrial species, have been in constant interaction with a high diversity of fungal micro-eukaryotes in their environments –consequently incorporating a diverse set of bacteria that mediate the growth of these fungi and indirectly serve as a protective trait against chytrids such as Bd and Bsal. Since two-lined salamander can also be present in the same environment as red-backed salamanders in our study site, this could explain the similar repertoire of protective bacteria. We hypothesize that the higher repertoire of protective bacteria in the microbial communities in our less Bd-susceptible species is an adaptation to mediate the growth of fungal micro-eukaryotes present in the soil that seek to colonize their skin.

The higher proportion of Bd-inhibitory bacteria as hubs along with the positive associations with these bacteria in the less Bd susceptible salamanders suggest that these beneficial bacteria and relationships are ecologically important in the host microbial community (e.g., supporting resistance to fungal infections). These bacteria may be cooperative and cross-feeding one another or not killing one another, signaling compounds (e.g., metabolites) through complex interactions that can enhance inhibition of pathogen growth and disease protection through distinct mechanisms (50, 51). We highlight the correlations with putative Bd-inhibitory bacteria, particularly with those considered hubs, to be an important part of the protective mechanisms against Bd (34). These microbial relationships could be essential to infer the influence of various interactions on the host protection against pathogens and health maintenance (26). For instance, microbial interactions could cause production of compounds with greater inhibition and prime the host immune system for faster activation of defense against pathogens (52, 53).

The adaptive immune system is completely dependent upon elements of the innate immune system for initiation of recognition and clearance of pathogens (54). Therefore, it is possible that the hub Bd-protective bacteria, particularly in the less Bd susceptible species, regulate or at least communicate in production of extracellular enzymes, metabolites and antimicrobial compounds or activation of the host immune system to protect host environment efficiently against invading microbes (55). Recent studies have demonstrated an important role of microbial metabolites in the regulation of host immune system. For instance, metabolites produced by gut microbiomes can activate host immune system through microbial sensors in intestinal epithelial cells after a pathogen infection (55).

Interestingly, five hub Bd-inhibitory bacterial ASVs in our data set matched with five culturable bacteria at 100% sequence similarity (Table S6), and these culturable Bd-inhibitory bacteria did not inhibit the growth of one another in coculture assays, providing support for the positive associations we observed of Bd-inhibitory hub bacteria with other bacteria in the co-occurrence networks. Further, the higher proportion of positive correlations of hub bacteria in our less Bd susceptible salamanders suggests a bacterial selection (positively associating similar lineage taxa to directly or indirectly pass genes), that is indicative of a healthy and well-functioning microbiome, contrary to a higher number of negative correlations (competitive exclusion) (56). Our data suggest that the role of Bd-inhibitory bacteria is determined both by the richness of these bacteria and also by the type of Bd-inhibitory hub bacteria and bacterial interactions, at least in terms of co-occurrence. Our results reveal that Bd-inhibitory bacteria play an important role in networks (e.g., hub bacteria), that microbial network “associations” are relevant, and emphasizes the importance of considering such Bd-protective hub bacteria and their potential interactions as possible mechanisms for protection against pathogens. Detailed functional studies of the bacterial associations we identified in our network analyses (e.g., protective bacterial strains of genera *Arthrobacter*, Pseudomonas and *Pedobacter*) will be a crucial step to better understand the biological significance of bacterial interactions related to Bd infection across our salamander species. Investigating bacterial interactions along with their interaction with micro-eukaryotes through a meta-transcriptomic approach would provide further insight into the functional interactions between microbes in the networks.

Our results improve our understanding of the complexity of host defense mechanisms to inhibit pathogens and mediate diseases which may have multiscale effects on salamanders’ ecological processes. It is imperative to keep advancing our knowledge about the relationship between skin microbiomes and AMPs among hosts to unravel mechanisms that may support amphibian’s coexistence with Bd in the environment and consequently improve our understanding of amphibian immune defense traits.

## MATERIALS AND METHODS

### Field sampling and study system.

Samples from salamanders were collected at the Smithsonian Conservation Biology Institute campus in Front Royal, VA. At this locality, we sampled 73 adult individuals from four species, *P. cinereus* (red-backed salamander, *n* = 35), *E. bislineata* (two-lined salamanders, *n* = 12), *D. monticola* (seal salamanders, *n* = 11) and *N. viridescens* (eastern newts, *n* = 25) in October 2018 from a single site. We also included *P. cinereus* (*n* = 5) and *N. viridescens* (*n* = 5) collected from Mountain Lake Biological Station, Pembroke, VA, in July 2017 (Table S1). The red-backed salamander *P. cinereus* is a fully terrestrial salamander that live in the leaf litter, under rocks, logs, and in small burrows. *Eurycea bislineata* is a semi-aquatic salamander that lives along and within stream banks covered by fallen leaves, logs and rocks. *Desmognathus monticola* is a semi-aquatic salamander inhabiting small streams and can be found under logs and rocks. Lastly, *N. viridescens* has a complex life cycle, but adults live in ponds, small lakes and marshes. These four salamander species are known to differ in their susceptibility to chytrid pathogens (Table 1).

After capturing a salamander, we rinsed it with sterile deionized water to remove sediments and transient microbes and placed it in a sterile whirl-pak bag. To obtain microbiome and chytrid samples (Table S1), we gently swabbed it 25 times (dorsal/ventral sides, front/back limbs, and tail; five strokes each). Swabs were placed in sterile 2 mL tubes containing 200 μL of 100% ethanol. Swab samples were then placed on dry ice for transport to the laboratory, where they were stored at −20°C.

For mucosome samples, we collected water bath samples from 51 individuals at Front Royal, VA, including all four species (Table S1), following the procedures as described in Woodhams et al. (7) with slight modifications. HPLC-grade water was added to the salamanders in the sterile whirl-pak bags, allowed the salamanders to sit in the water bath for 15 min and then poured the water bath into sterile tubes. We used 5 mL HPLC grade water for the small-bodied salamanders (*E. bislineata* and *P. cinereus*) and 10 mL for the medium-bodied salamanders (*N. viridescens* and *D. monticola*). For peptide samples, we collected skin secretions from two salamander species, *P. cinereus* (*n* = 4) and *N. viridescens* (*n* = 4) (Table S1). To obtain the peptide secretions, we used acetylcholine injections followed by a water bath (HPLC-grade water) in sterile whirl-pak bags for two individuals per species and used only water baths for the other two individuals per species. Acetylcholine-treated individuals were injected with 2.5 umol/gbw acetylcholine with an insulin needle in the flank region anterior to the back limb (57). Salamanders were allowed to sit in the water bath for 15 min and then poured the water bath into sterile tubes. We provide a descriptive analysis of these two different methods on AMP diversity, but to understand their efficacy in AMP collections is beyond the scope of this study. A larger sample size would be necessary to perform adequate statistical analyses to compare their effectiveness for AMP collections. Mucosome and peptide samples were stored on dry ice for transport to the laboratory. In the laboratory, samples were lyophilized and stored frozen until analysis.

### Mucosome assays against Bd and Bsal.

The magnitude of mucosome function (i.e., the ability to kill pathogens) was tested using *in vitro* assays with Bd or Bsal and mucus skin secretions collected from the salamanders. We grew Bd isolate JEL 404 and Bsal isolate AMFP-13/01 using standard procedures (7, 16). The Bd isolate is from a bullfrog and is an isolate similar to what salamanders in the eastern United States are exposed to in the field. We determined the number of remaining viable Bd and Bsal zoospores with a luminescence assay using a CellTiter-Glo 2.0 Cell Viability assay kit (Promega, G9242) following manufacture’s protocols and a POLARstar OMEGA microplate reader (BMG Labtech).

We performed assays by combining 50 μL of zoospores and 50 μL of mucosome solutions (100 μL total) in four replicate wells per salamander sample, within a 96-well plate. Prior to assays, lyophilized mucosome samples were reconstituted with 1 mL of miliQ water. The Bd and Bsal zoospores were both at a density of 1 × 10^6^ zoospores/mL (counted with a hemocytometer). Plates were incubated for 60 min at 21°C for Bd and 15°C for Bsal assays, and the amount of ATP (ATP) was measured using the luminescence assay (58). The mucosome samples may have also contained ATP, therefore multiple background controls were run. Within each 96-well plate we included four controls: sterile wash water (negative control 1), sterile chytrid growth media (negative control 2), heat-killed chytrid zoospores (heat-killed control), and viable Bd/Bsal in growth media combined with sterile wash water (positive control). The mucosome function was calculated against chytrid pathogens by dividing the corrected zoospore viability by the corrected positive-control Lum (light intensity) value and then subtract from 1 to get the mucosome function value—with higher values meaning greater killing ability. We excluded 10 of 51 of Bd trials and 1 of 51 Bsal trials from the analyses given that they showed a proportion viability higher than 100, which could be associated with unwanted materials in the samples (e.g., skin particles from skin shedding).

### Molecular methods and sequencing.

Genomic DNA was extracted from skin swab samples using the Biosprint blood and tissue kit (Qiagen, MD) with a pretreatment step for Gram-positive bacteria following manufacture’s protocol.

We used qPCR for the detection of Bd and Bsal infection following Blooi et al. (59) and using KlearKall Master mix (LCG). All swabs were tested in duplicate for Bd and Bsal. For Bd quantification, we used standards of 10^5^, 10^4^, 10^3^, 10^2^, 10, 1 and 0.1 ZGEs (zoospore genomic equivalents) developed from the Bd isolate JEL 427. For Bsal, a positive DNA extract control from isolate AMFP13/1 was used. If one replicate was positive and the other negative, the sample was run a third time. We considered a sample Bd+ or Bsal+ if it amplified at least twice, and for Bd if the threshold cycle was lower than that of the 0.1 ZGE standards.

We used a two-step PCR library prep and dual-index paired-end Illumina sequencing to sequence the skin microbiome of each individual (Supplemental methods). For the first PCR (amplicon PCR), we amplified an ∼380 bp region in the V3-V5 region of the 16S rRNA gene using the universal primers 515F-Y (GTGYCAGCMGCCGCGGTAA) and 939R (CTTGTGCGGGCCCCCGTCAATTC). Reactions were done in duplicate for each sample and included the negative extraction controls and negative PCR controls. Duplicate amplicon PCRs were pooled, and i5 and i7 adaptors were attached through index PCR. We cleaned post-PCR products with in-house Speed-beads (in a PEG/NaCl buffer), quantified DNA concentrations with a Qubit4 (Invitrogen, MA) and pooled final indexed samples together in equimolar proportion. The final library was size selected using a E-Gel Size Select II Agarose gel (Invitrogen, MA). The pooled library was sequenced on two Illumina MiSeq runs (v3 chemistry: 2 × 300 bp kit) at the Center for Conservation Genomics, National Zoo.

### Microbiome sequence processing.

We used R environment for sequence processing of demultiplexed reads. The package “dada2” (60) was used to perform quality filtering using standard filtering parameters (i.e., maxEE = 2), collapsed high quality reads into amplicon sequence variant (ASV) and removed chimeras. The bacterial taxonomy was assigned using the Ribosomal Database Project (RDP) trainset 16/release 11.5 (61). A phylogenetic tree of the bacterial ASVs was built using “align-to-tree-mafft-fasttree” pipeline from the “q2-phylogeny” plugin on QIIME2 (62).

We used package “phyloseq” (63) to import and merge the final ASV table, taxonomy table, bacterial phylogenic tree and metadata to create a phyloseq object to perform further analyses. Sequences classified as cyanobacteria/chloroplast, archaea, and unclassified phylum were removed and as well as singletons (i.e., ASVs with only one sequence read in one individual). We used package “decontam” (64) to remove potential contaminants using the method “either” with 0.1 and 0.5 thresholds for frequency and prevalence, respectively, and also removed any ASV that was found in more than two negative controls. For normalization of sequences counts, we performed variance-stabilizing normalization (cumulative sum scaling [CSS]) on the raw sequence counts using the package “metagenomeSeq” (65).

For the alpha diversity analyses, we included number of sequences as a covariate to control for sample sequencing depth. If sequencing depth was significant, analyses using a rarefied data set were conducted to confirm sequencing depth was not affecting the significance of the biological effect (as in Muletz et al. [66]). The sequencing depth did not influence our biological inference, so we report the statistics of the raw sequence counts in the results.

### Identification of putative Bd-inhibitory bacteria.

To identify the putative Bd-inhibitory ASVs, we first queried our ASV sequences against 1) a database consisting of sequences of anti-Bd bacteria identified a database of culturable anti-Bd bacteria identified from different amphibian species across the world (Antifungal Isolates Database; [67]). ASVs with 100% sequence identity match to those in the mentioned databases were retained following the methods outlined by Muletz-Wolz et al. (44) with the software Geneious version 20.1.2. Importantly, the function of microbial secondary metabolites can differ among Bd isolates (68), and secondary metabolite production and other functions are associated with 16S rRNA as phylogenetically conserved traits (69), while genes ascribing antifungal function were not examined and often differ among microbes. Thus, ascribing function of microbial communities is only an estimate, but is useful for between-group comparisons (70).

### Co-occurrence networks.

The role of putative Bd-inhibitory bacteria in structuring network communities were assessed using co-occurrence networks. Prior the construction of the networks, ASVs that were not detected more than 3 times in at least 20% of the samples were removed to reduce spurious correlations. We constructed the networks with the CoNet App for Cytoscape v 3.8.0 following their suggested network-building parameters (71). To determine if ASVs were important in structuring the community, defined as “hub taxa,” at least one of the three indicators of centrality were above the 75th percentile: degree, closeness centrality and betweenness centrality, as performed by Rebollar et al. (34). The calculation of the centrality indices and the visualization of networks were performed with Cytoscape v 3.8.0.

### Bacterial coculture assays.

We performed coculture assays with eight culturable Bd-inhibitory bacteria to determine if they had inhibitory effects against one another. Five of the culturable bacteria matched five hub Bd-inhibitory bacterial ASVs in our data set at 100% sequence similarity. The culturable bacteria were originally isolated from *P. cinereus* salamanders in Shenandoah NP, VA (72).

Each bacterial isolate was grown in monoculture in 1% tryptone broth for 48 h. We streaked the first bacterial strain horizontally on 1% tryptone plates with a sterile inoculating loop, waited for the bacteria to dry, then vertically streaked two other bacterial strains at each end of the horizontally streaked bacteria. This configuration was repeated until all bacterial strains had been challenged against all other bacterial strains (Supplemental methods). The coculture plates were examined for zones of inhibition daily for 7 days.

### Peptides.

We used core-shell hydrogel particles to harvest peptides from skin secretions of *P. cinereus* (*n* = 4) and *N. viridescens* (*n* = 4). Lyophilized samples were resuspended in 0.7–1.2 mL HEPES buffer (10 mM, pH 7.4) with protease inhibitor and divided each into two aliquots of equal volume. For harvesting, an aliquot of resuspended sample was added to 5 mg hydrogel particles in 0.7 mL Tris buffer (10 mM TrisCl, pH 7.4) that contained 60 pmol of each of three peptide standards (SMAP29, indolicidin, and LL37). The particle suspension was allowed to mix overnight at room temperature and the particles were then washed three times with Tris buffer. Bound peptides were eluted with 50% acetonitrile/0.1% trifluoroacetic acid, with the eluents then filtered and dried. The dried sample was then resuspended in 55 μL HEPES buffer. Peptides were reduced by adding 5 μL tris (2-carboxyethyl) phosphine hydrochloride solution and incubated for 25 min at 56°C, and then alkylated by adding 5 μL iodoacetamide solution and incubating for 30 min at room temperature. The sample was then acidified prior to being desalted and concentrated for LC-MS/MS analysis (Supplemental methods).

We analyzed the mass spectra (.RAW files) with PEAKS *de novo* sequencing software version X+ (Bioinformatics Solutions Inc.). Automated *de novo* sequencing was performed using a precursor mass error tolerance of 5 ppm, fragment mass error tolerance of 0.05 Da. We performed database-assisted sequencing using *de novo* sequence tags and a database of *P. cinereus* transcripts. Database identifications (with false discovery rate 1%) and *de novo* identifications with average residue local confidence ≥ 50% were BLAST searched against vertebrate proteins (taxid:7742) in the NCBI nonredundant protein database with settings adjusted for short input sequences (expect threshold 200,000; word size 2; matrix PAM30; no compositional adjustments). Only alignments with expect value ≤1E-3 were considered significant. Then, we performed feature-based label free quantification using PEAKSQ with a mass error tolerance of 5 ppm, retention time shift tolerance of 1 min, and automated reference and training sample selection. We calculated abundance based on feature area and performed normalization based on total ion chromatogram. PEAKSQ groups features matched between different samples based on mass and retention time agreement into feature vectors. Prior to performing further analysis, feature vectors were eliminated if they included a match to a control sample and had a charge of +1 (not considered peptide candidates and may result from contaminants (73). We considered peptides as AMP candidates when their gas-phase charge was between +3 to +8 and mass was > 1000, given the cationic character typically associated with antimicrobial peptides and the lower mass range observed for known amphibian antimicrobial peptides (74–76).

### Statistical analyses.

All statistical analyses were performed in the R environment version 3.6.3 (77).

### Bd infection prevalence and intensity in wild salamanders.

We examined the Bd infection prevalence and intensity among salamander species. Bd prevalence and the 95% confidence intervals (CIs) were calculated using the package ‘‘prevalence’’ (78). For Bd infection intensity, the mean and standard deviation (± SD) of the estimated number of zoospore genomic equivalents (ZGEs) were calculated.

### Mucosome function and its relationship with Bd infection prevalence and intensity in wild salamanders.

We compared mucosome function among salamander species using Generalized Linear Models (GLMs) with gaussian distribution and a log link function, and used *pos hoc* pairwise comparisons with False Discovery Rate correction (FDR) using the package “emmeans” (79). We investigated whether mucosome function differed between Bd and Bsal in the same salamander species using t-tests.

We used a hurdle model to determine if Bd infection prevalence and intensity predicts mucosome function (function hurdle in package “pscl”) (80) across salamander species. This model includes two-part process, first model infection status as a binomial process and then model infection intensity as a count process (poisson or negative-binomial process). For the infection intensity, the model uses data only from infected individuals. The hurdle model with negative binomial distribution had the best fit for the data compared to a hurdle model with Poisson distribution based on Akaike information criterion (AIC). A negative binomial model was also used with only *N. viridescens* samples (for which we had sufficient sample size) to investigate salamander species level effects on Bd infection intensity relationships to mucosome function.

### Microbiome characterization and the relationship of putative Bd-inhibitory bacteria diversity to Bd infection.

We first analyzed the entire bacterial community among salamander species. We provide a general description of taxonomic patterns. Then, the microbiome across species and environment in alpha and beta diversity was examined (Supplemental methods).

We compared the distribution of putative Bd-inhibitory ASVs among the four species using the package Vennerable (81). Hurdle models with negative binomial distribution were used to investigate if the richness and relative abundance of putative Bd-inhibitory bacterial ASVs predicts Bd infection prevalence and intensity. In addition, hurdle models were conducted to investigate a link of the bacterial ASVs richness from the whole bacterial community with Bd infection prevalence and intensity.

We performed GLMs with gaussian distribution and a log link function to evaluate relationships of richness and relative abundance of putative Bd-inhibitory bacterial ASVs with mucosome function with two data sets: i) all individuals (both Bd-infected and uninfected) and ii) Bd-infected individuals only. We also performed GLMs with gaussian distribution and a log link function to investigate a relationship of the bacterial ASVs richness from the whole bacterial community with Bd infection prevalence and intensity and used the two data sets from above.

### Putative Bd-inhibitory bacteria structuring network communities.

Independent tests (Chi-square tests and G-tests) were used to evaluate differences in the proportions of hub bacteria, Bd-inhibitory hub bacteria and bacterial associations between salamander species.

### Data availability statement.

Demultiplexed Illumina sequence data and associated metadata have been deposited in the National Center for Biotechnology Information Sequence Read Archive (www.ncbi.nlm.nih.gov/sra) under BioProject IDs: PRJNA659464.
