# Supplementary material for: Inhibitory Bacterial Diversity and Mucosome Function Differentiate Susceptibility of Appalachian Salamanders to Chytrid Fungal Infection
Source: Appl Environ Microbiol. 2022 Mar 29;88(8):e01818-21. doi: 10.1128/aem.01818-21 (PMC9040618; doi:10.1128/aem.01818-21)
Supplement: Supplemental file 1 — Fig. S1 to S3, Tables S1 to S4 and S6, and supplemental methods. Download aem.01818-21-s0001.pdf, PDF file, 0.5 MB [file aem.01818-21-s0001.pdf]

Supporting information for:
Inhibitory bacterial diversity and mucosome function differentiate susceptibility of Appalachian salamanders to chytrid fungal infection

Randall R. Jiménez, Amy Carfagno, Luke Linhoff, Brian Gratwicke, Douglas C. Woodhams, Liana Soares Chafran, Molly C. Bletz, Barney Bishop, Carly R. Muletz-Wolz

This file includes:
Figure S1. Relationship between Bd infection intensity and mucosome function (measure of the pathogen-killing ability of skin mucus samples) on *N. viridescens*. The continuous line indicates the predicted fit and the shaded areas are 95% confidence intervals.
Figure S2. Skin microbiome of four salamander species
Figure S3. Alpha diversity of the skin bacterial communities from four salamander species

Table S1 Total number of individuals per species and locality for which samples were analyzed to conduct microbiome, mucosome and peptides
Table S2 Summary of GLMs predicting alpha diversity according to salamander species, sampling area and number of sequences
Table S3 Summary of PERMANOVA models of unweighted and weighted UniFrac according to host species and sampling area
Table S4 PERMANOVA pairwise comparisons of unweighted and weighted UniFrac between host species
Table S6 Culturable Bd-inhibitory bacterial strains used in co-culture assays. Bd inhibition values are from Muletz-Wolz et al. (2017).

Supplementary methods for amplicon sequencing of skin bacterial communities
Supplementary methods for characterization of skin bacterial communities
Supplementary methods for bacterial co-culture assays
Supplementary methods for analysis of antimicrobial peptides (AMPs)

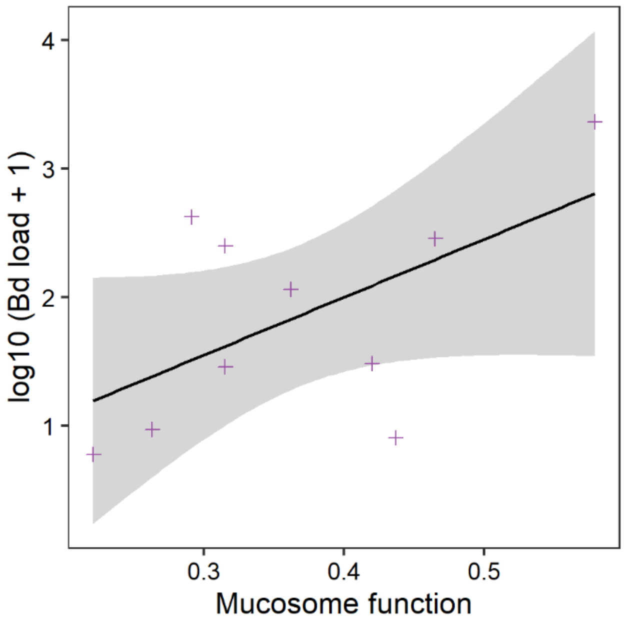

Figure S1 Relationship between Bd infection intensity and mucosome function (measure of the pathogen-killing ability of skin mucus samples) on *N. viridescens*. The continuous line indicates the predicted fit and the shaded areas are 95% confidence intervals.

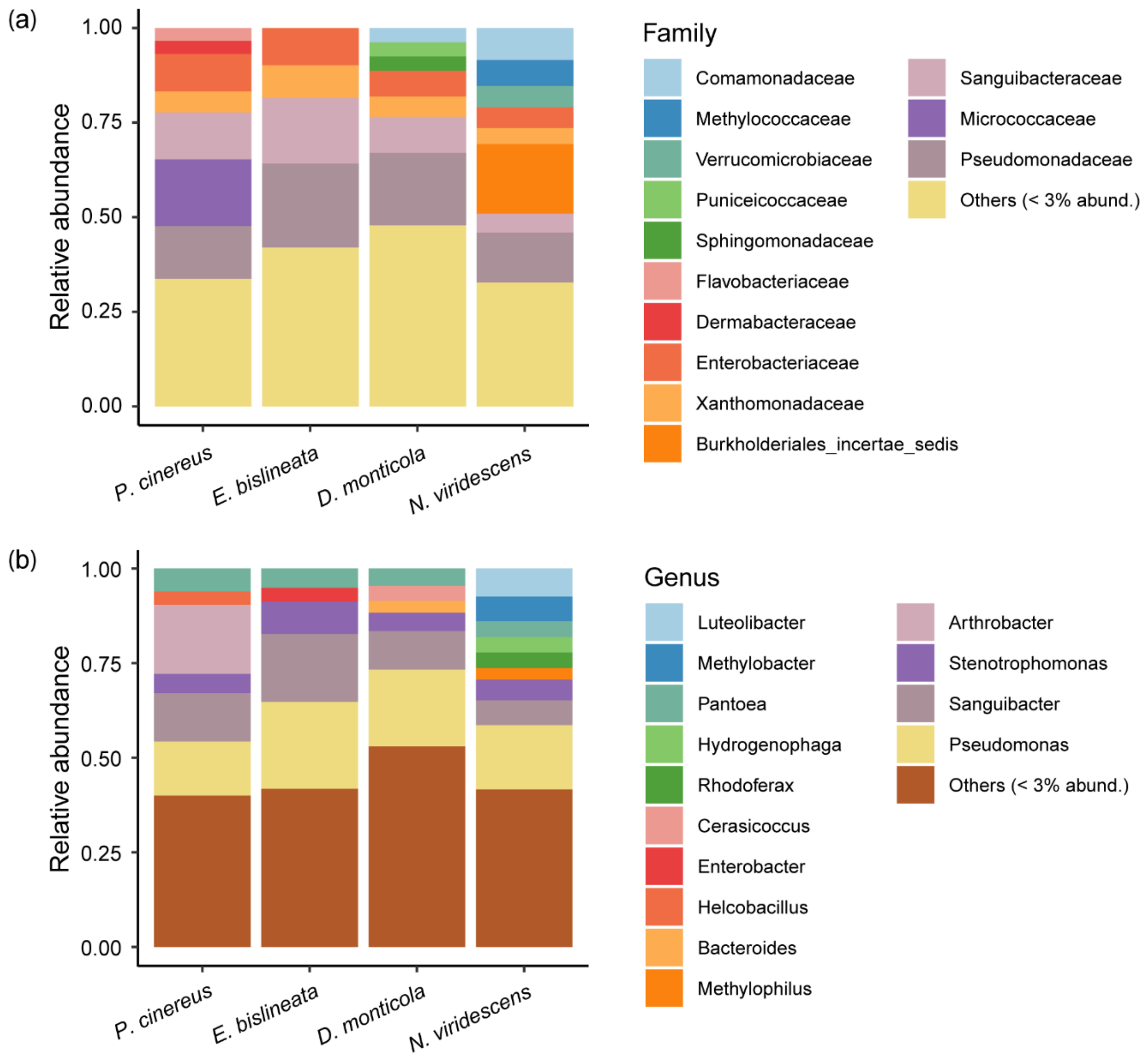

Figure S2 Skin microbiome of four salamander species. ***(a)*** Relative abundance of skin bacterial taxa at the Family level across species. ***(b)*** Relative abundance of skin bacterial taxa at the Genus level across species. Rare ASVs (relative abundances < 3%) were clustered together.

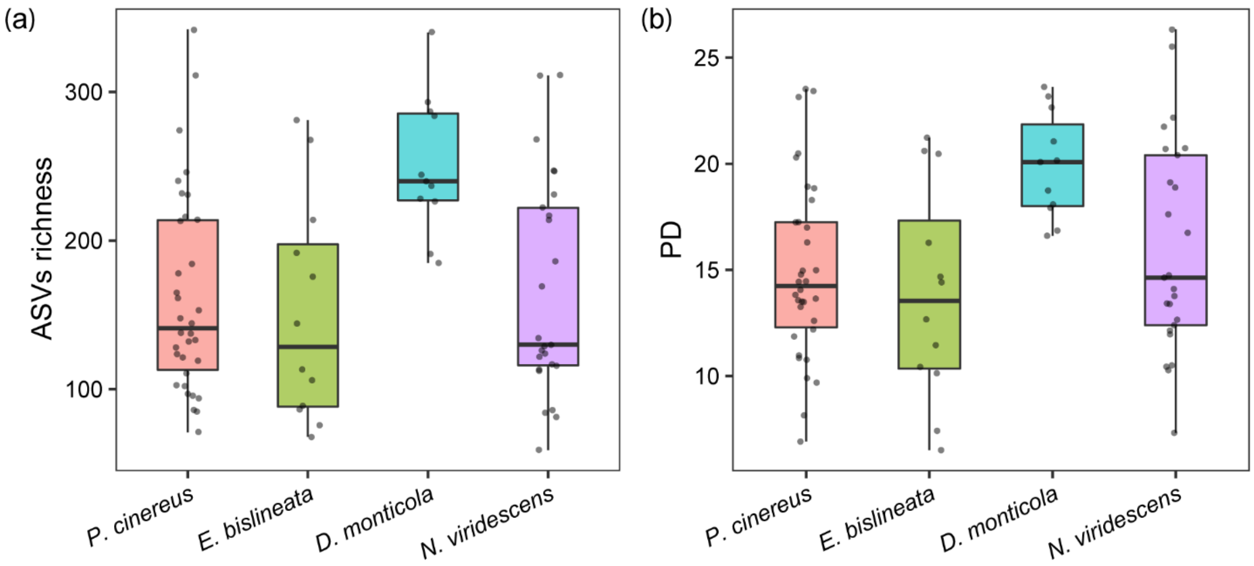

Figure S3 Alpha diversity of the skin bacterial communities from four salamander species. (*a*) ASVs richness ($p < 0.001$); (*b*) Faith's phylogenetic diversity (PD) ($p = 0.013$). Each gray point represents the bacterial skin community of an individual sample.

Table S1. Total number of individuals per species and locality for which samples were analyzed to conduct microbiome, mucosome and peptides. Numbers in parenthesis are the total number of samples collected; some samples were excluded due to apparent differences in sample readings (mucosome) or sample preparation (peptides).

Genus	Species	Locality	Total N sampled (microbiome + chytrid)	N mucosome	N peptides profiled
Plethodon	*cinereus*	Front Royal, VA	30	14 (16)	4 (14)
Plethodon	*cinereus*	Mt. Lake, VA	5	--	--
Eurycea	*bislineata*	Front Royal, VA	12	10 (12)	--
Desmognathus	*monticola*	Front Royal, VA	11	7 (11)	--
Notophthalmus	*viridescens*	Front Royal, VA	20	11 (12)	4 (8)
Notophthalmus	*viridescens*	Mt. Lake, VA	5	--	--
	**Total:**		83	42	8

Table S2. Summary of GLMs predicting alpha diversity according to salamander species, sampling area and number of sequences.

Variables	LR Chisq (χ^2)	Df	*p*-value
**Number of observed ASVs**			
Species	15.56	3	0.001
Sampling area	6.03	1	0.014
Number of sequences	22.83	1	< 0.001
**PD**			
Species	9.04	3	0.013
Sampling area	6.13	1	0.03
Number of sequences	18.15	1	< 0.001

Table S3. Summary of PERMANOVA models of unweighted and weighted UniFrac according to host species and sampling area.

Variables	SumsOfSqs	MeanSqs	F.Model	R^2	*p*-value
**Unweighted UniFrac**					
Sampling area	0.62	0.62	6.78	0.06	0.001
Host species	3.04	1.01	11.06	0.28	0.001
Residuals	7.16	0.09		0.66	
Total	10.82			1.00	
**Weighted UniFrac**					
Sampling area	0.26	0.26	5.11	0.03	0.004
Host species	3.55	1.18	22.76	0.45	0.001
Residuals	4.06	0.05		0.51	
Total	7.88			1.00	

Table S4. PERMANOVA pairwise comparisons of unweighted and weighted UniFrac between host species. Significant *p*-values (<0.05) are shown in bold.

**Comparison**	**R^2**	***p*-value**	**Adjusted *p***
**Unweighted UniFrac**			
P. cinereus – E. bislineata	0.07	0.001	**0.006**
P. cinereus – D. monticola	0.09	0.001	**0.006**
E. bislineata – D. monticola	0.12	0.002	**0.012**
N. viridescens – P. cinereus	0.25	0.001	**0.006**
N. viridescens – E. bislineata	0.22	0.001	**0.006**
N. viridescens – D. monticola	0.27	0.001	**0.006**
**Weighted UniFrac**			
P. cinereus – E. bislineata	0.10	0.002	**0.012**
P. cinereus – D. monticola	0.17	0.001	**0.006**
E. bislineata – D. monticola	0.08	0.117	0.702
N. viridescens – P. cinereus	0.48	0.001	**0.006**
N. viridescens – E. bislineata	0.38	0.001	**0.006**
N. viridescens – D. monticola	0.35	0.001	**0.006**

Table S6. Culturable Bd-inhibitory bacterial strains used in co-culture assays. Bd inhibition values are from Muletz-Wolz et al. (2017).

**Bacterial strain**	**Bd inhibition in vitro**	**ASV Match**	**Bd-inhibitory hub taxa in species**
Pseudomonas RSB5.2	8%	ASV35	*P. cinereus, E. bislineata*
Pseudomonas RSB5.4	99%	ASV37	*P. cinereus, E. bislineata, D. monticola*
Duganella SFB1.10.B69	12%	ASV86	*E. bislineata*
Stenotrophomonas THA2.2	100%	ASV15	*P. cinereus, N. viridescens*
Microbacterium THC2.6	7%	ASV30	*P. cinereus, N. viridescens*
Mitsuaria LSB4.1	27%	ASV236	--
Acinetobacter THA6.5	32%	--	--
Pedobacter SFA3.14	88%	--	--

Supplementary methods for amplicon sequencing of skin bacterial communities

For the amplicon PCR, we performed PCR in 25 ul reactions using 12.5 μL of 2X KAPA HiFiHotStart ReadyMix (Kapa Biosystems, USA), 0.3 uM forward and reverse primers, 20 ug of BSA, and 3 μL of DNA. PCR conditions were: 95° C for 3 min, followed by 25 cycles of 98° C for 20 sec, 62° C for 15 sec, 72° C for 15 sec, and a final extension (72° C for 1 min). Then, we performed index PCR in 25 μL reactions using 12.5 μL of 2X Kapa HiFi HotStart ReadyMix (Kapa Biosystems, USA), 0.8 uM i5 and i7 primers, and 3 μL of DNA. PCR conditions were: 95°C for 3 minutes, 98°C for 20 sec, 62°C for 15 sec, and 72°C for 15 sec, and a final extension (72°C for 1 min).

Supplementary methods for characterization of skin bacterial communities

We first provide a general description of taxonomic patterns. We generated stacked bar charts at the family, and genus levels to visualize differences among skin bacterial taxa across salamander species. These charts were produced using the R packages "phyloseq" (McMurdie and Holmes, 2013) and "ggplot2" (Wickham, 2016). Then, we examined the microbiome across species at three levels, alpha diversity, beta diversity and Bd-inhibitory bacterial richness.

To examine alpha diversity of the skin bacterial communities we calculated two metrics: Faith's phylogenetic diversity (PD) and total number of observed ASVs (ASVs richness) for each sample using the R package "picante" (Kembel et al., 2010). We tested whether the alpha diversity metrics varied across host species and locality by fitting GLMs with a gamma distribution and a log link function. We included number of sequences as a covariate to control for sample sequencing depth. We performed *post hoc* pairwise comparisons with False Discovery Rate correction using the R package "emmeans" (Lenth, 2019).

To examine beta diversity of skin bacterial communities we calculated two metrics: unweighted UniFrac and weighted UniFrac (Lozupone et al., 2011) from normalized sequence counts using the R package "phyloseq". We tested whether the beta diversity metrics varied across host species and locality using PERMANOVAs (function *adonis2* in R package "vegan"). We performed pairwise PERMANOVAs for significant effects with Bonferroni corrections. We visualized differences in beta diversity using the first three axis of the Non-Metric Multidimensional Scaling (NMDS) using "phyloseq".

References

Kembel, SW, Cowan, PD, Helmus, MR., Cornwell, WK, Morlon, H, Ackerly, DD, Blomberg, SP, & Webb, CO. 2010. Picante: R tools for integrating phylogenies and ecology. Bioinformatics 26(11): 1463–1464.

Lenth, R. 2019. Emmeans: Estimated marginal means, aka least-squares means. R package version 1.3.2. Available online at: https://CRAN.R-project.org/package=emmeans

Lozupone C, Lladser ME, Knights D, Stombaugh, J, Knight R. 2011. UniFrac: An effective distance metric for microbial community comparison. The ISME Journal, 5(2): 169–172.

McMurdie, P. J., and Holmes, S. 2013. phyloseq: An R package for reproducible interactive analysis and graphics of microbiome census data. PLOS One: 8(4).

Wickham, H. 2016. ggplot2: Elegant Graphics for Data Analysis. Springer-Verlag New York.

Supplementary methods for bacterial co-culture assays

We grew each bacterial isolate in monoculture in 1% tryptone broth for 48 hours. We streaked the first bacterial strain horizontally on 1% tryptone plates with a sterile inoculating loop, waited for the bacteria to dry, then vertically streaked two other bacterial strains at each end of the horizontally streaked bacteria. We repeated this configuration until all bacterial strains had been challenged against all other bacterial strains. We examined the co-culture plates for zones of inhibition daily for seven days.

Supplementary methods for analysis of antimicrobial peptides (AMPs)

LC-MS/MS: Peptides were resuspended in 0.1% formic acid for analysis on a Thermo Scientific Orbitrap Fusion Tribrid (Thermo Fisher Scientific, MA). Peptides were separated using a reversed-phase 200 µm diameter x 25 cm long polystyrene divinylbenzene PepSwift monolithic capillary HPLC column. Mobile phase A consisted of 0.1% aqueous formic acid and mobile phase B consisted of 0.1% formic acid in 80% acetonitrile. After sample injection, the column was washed for 2 min with A; peptides were eluted using a linear gradient from 5 to 50% B over 60 min followed by ramping to 100% B for an additional 2 min. Flow rate was 1250 nL/min. The Orbitrap Fusion was operated in a data-dependent mode in which one full MS scan (resolution 60,000) from 300–1500 Da using quadropole isolation (1.4 Da) and 50 ms maximum injection time was followed by MS/MS scans in which the most abundant molecular ions were dynamically selected by Top Speed. Electron transfer dissociation (ETD) fragmentation and higher-energy collisional dissociation (HCD) fragmentation were performed separately to generate complementary MS/MS spectra. Fluoranthene was the electron-transfer reagent with charge-state-dependent ETD reaction time and 50 ms maximum injection time. For HCD, normalized collision energy was 27% and maximum injection time was 50 ms. ETD and HCD fragments were detected in the Orbitrap (resolution 15,000).
